# Involvement of Eukaryotic Small RNA Pathways in Host Defense and Viral Pathogenesis

**DOI:** 10.3390/v5112659

**Published:** 2013-10-30

**Authors:** Julie Hicks, Hsiao-Ching Liu

**Affiliations:** Department of Animal Science, North Carolina State University, Raleigh, NC 27695, USA; E-Mail: jahicks3@ncsu.edu

**Keywords:** small RNA, immunity, pathogens

## Abstract

Post-transcriptional gene regulation by small RNAs is now established as an important branch of the gene regulatory system. Many different classes of small RNAs have been discovered; among these are short interfering RNAs (siRNAs) and microRNA (miRNAs). Though differences in the processing and function of small RNAs exist between plants and animals, both groups utilize small RNA-mediated gene regulation in response to pathogens. Host encoded miRNAs and siRNAs are generated from viral RNA function in host defense and pathogenic resistance in plants. In animals, miRNAs are key regulators in both immune system development and in immune function. Pathogens, in particular viruses, have evolved mechanisms to usurp the host’s small RNA-mediated regulatory system. Overall, small RNAs are a major component of host defense and immunity in eukaryotes. The goal of this review is to summarize our current knowledge of the involvement of eukaryotic small RNA pathways in host defense and viral pathogenesis.

## 1. Small RNA Biogenesis

**Figure 1:**
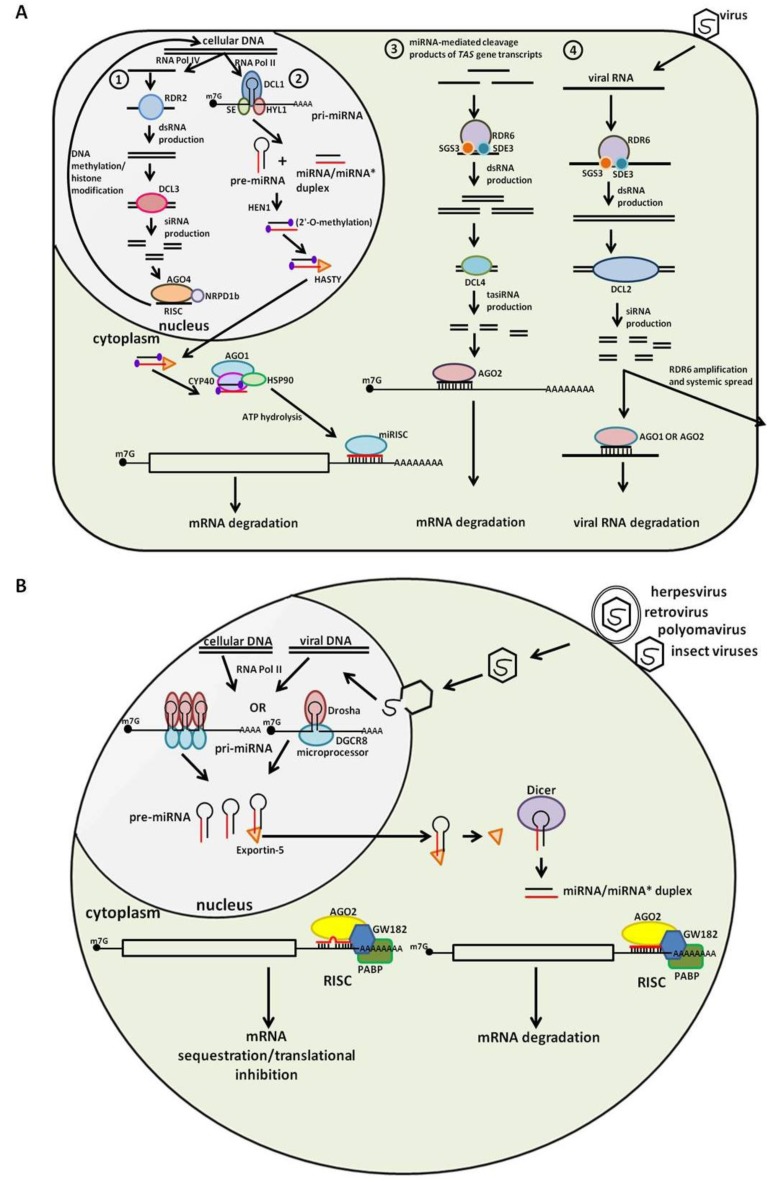
Endogenous small RNA pathways involved in host defense and viral pathogenesis in plants and animals. (**A**) Small RNA biogenesis pathways mediated by the four Dicer-like endonucleases (DCLs) in plant cells. (1) DCL3 generates short interfering RNAs (siRNAs) from transcripts produced via RNA Pol IV-dependent transcription. These siRNAs then form a RNA-induced silencing complex (RISC) with Argonaute 4 (AGO4), which mediates chromatin modifications at complementary genomic DNA sites. (2) Plant primary-microRNA (pri-miRNA) transcribed by RNA Pol II is processed into a miRNA/miRNA* duplex by DCL1. The duplex is then transported to the cytoplasm by Hasty, a homolog of animal exportin-5, where the mature miRNA associates with Argonaute 1 (AGO1) to form a miRISC (miRNA-induced silencing complex). miRISC facilitates the degradation of targeted mRNAs. (3) RNA dependent RNA polymerase 6 (RDR6) produces dsRNAs from miRNA-mediated cleavage products of *TAS* gene transcripts. These dsRNAs are then processed into trans-acting siRNAs (tasiRNAs) by DCL4. Mature tasiRNAs form a RISC with AGO2 to mediate mRNA suppression. (4) Viral RNAs are utilized by RDR6 to produce dsRNAs, which are then processed into siRNAs by DCL2. These viral siRNAs undergo a second round of RDR6 amplification and are transported to peripheral sites, where they form RISCs with either AGO1 or AGO2. These RISCs then degrade viral RNA as part of the anti-viral response; (**B**) MicroRNA biogenesis pathway of animal cells. Pri-miRNAs transcribed from either cellular DNA or viral DNA via an RNA Pol II mechanism are processed into precursor hairpin molecules (pre-miRNA) in the nucleus. Pre-miRNAs are transported to the cytoplasm, where they are further processed into the mature miRNA, which associates with AGO2 and a several accessory proteins to form RISC. RISC then facilitates the suppression of mRNA expression.

There are several classes of small RNA families, and of these, short-interfering RNAs (siRNAs) and microRNAs (miRNAs) are the major small RNA groups associated with eukaryotic immunity. The biogenesis pathways of siRNAs and miRNAs are well conserved and greatly overlap (Figure 1). In the generation of both groups, a precursor molecule is processed into a short double-stranded RNA (dsRNA) duplex by a member of the Dicer family of endonucleases. However, in animals, primary miRNA transcripts, which can contain multiple miRNAs, are initially processed into single precursor miRNAs by the endonuclease, Drosha. For siRNAs, the precursor molecule for Dicer processing is a longer dsRNA, while the miRNA precursor is an RNA hairpin [1]. Currently, mammals are thought to have only a single Dicer; flies and worms encode two Dicers, while plants can have up to four Dicer-like endonucleases (DCLs), depending upon the species. In addition to its endonuclease activity, Dicer also functions as a helicase and separates duplex RNA into two single-stranded small RNA molecules [2]. One strand, often termed the guide strand, interacts with members of the Argonaute protein family to form the RNA-induced silencing complex, or RISC, while the other strand, called the passenger or star strand, is usually degraded. RISC then facilitates the interaction of the small RNA with its target sequence, resulting in silencing or suppression of gene expression, by one of several mechanisms, which will be briefly reviewed below. 

## 2. Plant Small RNA: An Overview

Plants produce a variety of small RNA species, which possess three main functions: (1) regulation of transposon activity; (2) pathogenic defense; and (3) regulation of intrinsic pathways, such as development and the response to environmental stresses [3]. Plants produce a variety of endogenous small RNAs. In general, plants encode four DCLs, each of which process distinct small RNA (sRNA) classes, but also share some overlapping or redundant functions (Figure 1B). DCL1 is involved in the generation of 21–22 nt sRNAs. DCL1 preferentially associates with hairpin precursor RNAs [3]. RNA processing by DCL2 results in small 22 nt siRNAs. DCL3 generates sRNAs of 24 nt, while DCL4 is involved in the generation of 21 nt sRNAs from precursors consisting of long prefect complementary dsRNA precursors [3]. DCL1 is mainly involved in miRNA processing and possesses functions analogous to both Dicer and Drosha in animals [4], while DCL2 and DCL4 are the major siRNA processors [5]. DCL2 siRNAs mainly function in host defense against viruses, though in the absence of DCL2, DCL4 can also produce antiviral siRNAs [6]. DCL3 siRNAs mainly regulate transposon activity and chromatin modification [4]. DCL4 processing is mainly reserved for trans-acting siRNAs (tasiRNAs). DCL4 tasiRNAs are mainly induced by and regulate the response to environmental stresses, such as drought. Plants encode approximately ten different Argonaute proteins (Agos), and similar to DCLs, these Agos have both distinct and overlapping functions (reviewed by [7]). AGO1 is the major Ago protein in miRNA RISCs. AGO2 interacts with tasiRNA generated from DCL4, while AGO4 is the main ago mediator of DCL3-processed siRNA function. A summary of RNA interference (RNAi) pathways in plants is shown in Figure 1A.

## 3. Animal Small RNA: An Overview

Animals also produce several distinct small RNAs, though not as many as the wide range found in plants. The major endogenous class of small RNAs involved in regulating the immune response and immune system development in animals consists of miRNAs. The other types of endogenous small RNAs are mainly involved in the regulation of development, particularly in embryos. In animals miRNA genes are mainly located in either the introns of protein-coding genes or are located in intergenic regions. Intergenic miRNAs are under the control of their own promoters, while intronic miRNAs most often are under the control of their host gene, though some intronic miRNAs have been found to have their own promoter. Though single miRNAs exist, miRNAs are often found in clusters in animal genomes. Many miRNAs are initially expressed as a single primary transcript, consisting of multiple pre-miRNA hairpins. These hairpins are individually released by Drosha. After Drosha processing, the miRNA hairpins are transported from the nucleus to the cytoplasm by an exportin protein. Once in the cytoplasm, the hairpin is further processed by Dicer, and the mature miRNA guide sequence is then loaded onto the RISC [2] (Figure 1B).

## 4. Plant Immunity and Small RNA

In contrast to animals, a major plant antiviral defense mechanism involves RNA silencing. Upon infection, viral dsRNA is recognized by DCLs and is cleaved into viral siRNAs (vsiRNAs) that are 21–24 nt in length [8]. These vsiRNAs are then amplified by host RNA-dependent RNA polymerases. The secondary population of siRNAs is then transported throughout the plant as part of the antiviral response. Of the four plant DCLs, DCL2 appears to be the major DCL associated with siRNA-mediated viral defense, though DCL4 may also contribute. 

Plant PRRs (pathogen recognition receptors) are evolutionarily related to Toll-like receptors in animals [9]. They are typically receptor-like kinases. The resistance proteins involved in effector-triggered immunity are structurally analogous to Nod-like receptors in mammals. These resistance genes are intracellular molecules that function to survey the cytosol for pathogen-derived molecules. In plants, the chloroplast is also important in the immune defense. This defense is also involved in the production of oxidative species that are associated with hyper sensitive cell death response. The PAMPs (pathogen-associated molecular patterns) recognized by plant immune proteins are not necessarily pathogenic and are often structural [10]. For example, in *A. thaliana*, the PRR, FLAGELLIN SENSING 2, is a well-known receptor-like kinase involved in the detection of bacterial flagellin [10]. In addition to PRR/PAMP-triggered immunity, another major defense mechanism in plants is effector-triggered immunity (ETI). In ETI, a family of proteins, termed resistance or R proteins, recognize bacterial and viral antigens, which then triggers an immune response [11]. PRR/PAMP-triggered immunity and ETI are both regulated by and regulate small RNAs [12]. For example, bacterial infections can alter miRNA expression, including *miR-393*, *miR-167*, *miR-160* and *miR-825* [13]. These miRNAs regulate the expression of auxin signaling genes, as well as biotic stress-related genes, which are associated with PAMP signaling. Also important in ETI are host-encoded resistance genes, termed nucleotide binding sites, and leucine-rich repeat resistance proteins, or NBS-LRR proteins. In healthy plants the expression of NBS-LRR proteins is regulated by *miR-482* [14]. However, when a plant becomes infected with either a virus or bacterium, *miR-482* expression is downregulated, which then allows for increased expression of NBS-LRR proteins and induction of ETI. Melons express a member of the NBS-LRR protein family, Vat, which participates in the resistance to aphids [15]. MiRNA profiling during aphid infestations of resistant and susceptible plants revealed that, in general, miRNAs are upregulated in the resistant plants and downregulated in susceptible plants [15]. Many of the miRNAs upregulated in resistant plants regulate biotic and abiotic stress response genes. This data suggests that miRNA regulation is likely associated with disease resistance in plants. SiRNAs, such as *nat-siRNAATGB2* and *Atl-siRNA-1*, are induced upon pathogenic infection and regulate the expression of several resistance genes [16]. In plants, siRNAs also play a role in the silencing of transposition by facilitating the methylation of transposable elements [6]. However, this mechanism requires the cooperation of histone deacetylase 6 (HDA6). In *HDA6* mutant plants, siRNAs accumulate and induce *de novo* methylation, but this methylation does not result in the suppression of gene expression [6]. In the plant germline, there exists a special class of siRNA, which is termed epigenetically-activated 21 nt siRNA, or easiRNA [17]. EasiRNAs are involved in the regulation of numerous host defense genes via epigenetic mechanisms.

## 5. Animal Immunity and Small RNA

The involvement of miRNAs in the regulation of immunity and the immune response is well characterized in animals. Analysis of miRNA expression and regulation in lymphocytic progenitors and in differentiated lymphocytes found that, on average, each of these cell types expresses ~100 different miRNAs [18]. CHIP-seq (chromatin immunoprecipitation sequencing) analysis further revealed that in lymphocytic progenitor cells, lymphocyte-specific expression of miRNAs is under epigenetic regulation and that these miRNAs are only fully expressed upon differentiation. In all, ~50 miRNAs are upregulated upon lymphocyte maturation. MiRNA expression profiling of human primary macrophages found 119 expressed miRNAs. Several of the most highly expressed of these miRNAs, such as *miR-146a* and *miR-212*, are known to have immune-related functions [19]. 

Treatment of the acute myeloid leukemia cell line, THP-1 cells, with phorbol myristate acetate (PMA) to induce differentiation, was found to induce the expression of 23 PMA-regulated miRNAs [20]. Further analysis revealed that four of these miRNAs, *miR-155*, *miR-222*, *miR-424* and *miR-503,* work in concert to induce cell-cycle arrest and differentiation of myeloid progenitor cells.

Several immune miRNAs have been well characterized; among these is *miR-155*. There are currently over 100 studies published on *miR-155*, the majority of which reveal its involvement in modulating the immune response and in immune cell proliferation. CD8^+^ T-cells deficient for *miR-155* expression have reduced capacity to control viral infections [21]. This reduced efficacy of CD8^+^ T-cells lacking *miR-155* was linked to the accumulation of the *miR-155* target, SOCS-1 (suppressor of cytokine signaling-1), which ultimately reduces STAT5 (signal transducer and activator of transcription 5) signaling [21]. *MiR-155* expression is induced in murine macrophages following treatment with either polyriboinosinic:polyribocytidylic acid or IFN-β, suggesting a role for *miR-155* in the inflammatory response [22]. This increase in *miR-155* expression is facilitated by TLR (toll-like receptor) signaling. *MiR-155* was identified as part of an IFN signaling feedback system, which is MyD88 and NFκB dependent [23]. Two other TLR-regulated miRNAs have also been identified, *miR-21* and *miR-146a* [24]. *MiR-155* expression can be induced by TLR2, TLR3, TLR4 or TLR9; *miR-146a* is responsive to TLR2-TLR5, and *miR-21* is induced by TLR4 [25]. TLR signaling, in turn, can be regulated by miRNAs. *TLR3* and *TLR4* contain *miR-223* binding sites [25]. *TLR4* is also a target of *let-7i* and *let-7c*, while *TLR2* is regulated by *miR-105.*

Multiple studies have revealed a reciprocal relationship between NFκB and miRNAs, i.e., NFκB signaling regulates the expression of multiple miRNAs, and in turn, miRNAs regulate the expression of multiple members of NFκB signaling. NFκB signaling upregulates the expression of the pro-inflammatory cytokine, IL-6, by reducing *let-7* expression, an *IL6* regulator [26]. Conversely, NFκB upregulates the expression of both *miR-146* isoforms, which reduce the expression of IRAK1/TRAF6, which then decreases NF-κB activity [27].

## 6. Pathogenic Manipulation of Small RNA Pathways

The majority of currently known viral miRNAs are encoded by herpesviruses, which encode between three (Herpes B virus) to 68 (Rhesus lymphocryptovirus) mature miRNAs [28]. Several studies have suggested that these herpesviral miRNAs are key regulators of viral latency. Examples of herpesvirus-encoded miRNAs and their function(s) in viral latency and transformation are given in Table 1.

**Table 1:** Examples of virally-encoded miRNAs. KSHV, Kaposi’s sarcoma-associated herpesvirus; HSV1, herpes simplex virus 1; MDV, Marek’s disease virus; HCMV, human cytomegalovirus; EBV, Epstein-Barr virus; BLV, bovine leukemia virus; MICB, major histocompatibility complex class I polypeptide-related sequence B; ICP0, infected cell protein 0.

Virus	microRNA	Effect	Ref.
HSV1	*miR-H2-3p*	Targets viral *ICP0*; regulates latency	[29]
KSHV	*miR-K12-4-5p*	Targets *RBL2*; modulates epigenetic regulation	[30]
KSHV	*miR-K12-7*	Targets viral *RTA*; regulates latency	[30,31,32, 33]
KSHV	miR-K9*	Targets viral *RTA*; regulates latency	[33]
HCMV	*miR-UL112-1*	Targets host restriction factor BclAf1; enhances viral replication	[34,35]
KSHV	*miR-K12-11*	Hosts *miR-155* ortholog; regulates cell proliferation	[35]
MDV1	*miR-M4*	Hosts *miR-155* ortholog; regulates cell proliferation	[36]
EBV	*miR-BART1-3p, miR-BART5-5p, miR-BART22-3p*	orthologs to host miR-29a/b/c, miR-18a/b andmiR-520d/miR-524-5p, respectively; regulate apoptosis and the cell cycle	[37]
BLV	*blv-miR-B4*	Hosts *miR-29* ortholog; contributes to viral-induced lymphoma	[38]
KSHV, EBV, HCMV	*miR-K12-7, miR-BART2-5p, miR-UL112*	Target host MICB; virus immune evasion	[39]

Many herpesvirus miRNAs are located in genomic regions associated with viral latency. The latency associated transcript (*LAT*) of herpes simplex virus 1 (HSV1) encodes four miRNAs [29]. One of these miRNAs, *miR-H2-3p*, is likely involved in latency regulation by targeting the viral gene, *ICP0*, an immediate-early gene that is associated viral replication and reactivation [29]. Twelve miRNAs located in the latency-associated region (LAR) of Kaposi’s sarcoma-associated herpesvirus (KSHV) are expressed during viral latency [30]. These miRNAs have also been linked to viral latency and reactivation. A host gene involved in the epigenetic regulation of KSHV, *RBL2*, as well as the viral gene, *RTA* (replication and transcription activator), a reactivation regulator, are targets of KSHV miRNAs [30]. RTA is an immediate early gene considered a lytic switch for reactivation of latent KSHV. The KSHV-encoded *miR-K12-7* is able to regulate *RTA* expression via a recognition site within its 3' UTR (untranslated region) [31]. A KSHV deletion mutant lacking 10 KSHV miRNAs, including *miR-K12-7*, displayed increased expression of lytic genes, including RTA [32]. RTA is also likely regulated by another KSHV-encoded miRNA, termed miR-K9* [33]. Disruption of miR-K9* function in KSHB latently infected cells resulted in increased viral reactivation [33]. Together, these studies suggest that virally-encoded miRNAs are an important aspect in the maintenance of KSHV latency. MicroRNA sequence analysis of KSHV tumors revealed that some KSHV-encoded miRNAs are more susceptible to mutations than others [40]. The KSHV-encoded miRNAs, *miR-K12-1*, -*3*, -*8*, -*10*, -*11* and -*12* were conserved between patients, while *miR-K12-2*, -*4*, -*5*, -*6*, -*7* and -*9* had much more inter-patient sequence variability, which could impact their functionality. Human cytomegalovirus (HCMV), which encodes 17 mature miRNAs, expresses a miRNA, termed *miR-UL112-1*, during the late stages of infection, which targets the host restriction factor, *BclAf1* [34]. This miRNA targeting and subsequent reduction in BclAf1 expression enhances viral gene expression and replication. BclAf1 has been shown to target the viral protein, IE1, as part of the host anti-viral response to HCMV [36]. These results suggest that HCMV *miR-UL112-1* is involved in viral evasion of the host immune response. Multiple miRNAs have been identified in the genome of Marek’s disease virus (MDV), a well-known herpesvirus of poultry. Oncogenic MDV strains, classified as MDV serotype 1 (MDV-1), encode 14 precursor miRNAs, which produce 26 mature miRNAs [28]. Expression studies have revealed that MDV-1 miRNAs are differentially expressed during infections with strains of varying virulence [36]. These studies have also shown that miRNAs located near *MEQ*, a known MDV oncogene, are expressed at higher levels in infections with highly virulent strains compared to less virulent strains. This differential expression has been linked to a polymorphism, which is the likely promoter of these viral miRNAs. 

Several herpesviruses encode an ortholog to a host miRNA, *miR-155*, including KSHV and MDV. Currently, *miR-155* is one of the most studied miRNAs, in part because it has been linked to lymphocyte development and is often upregulated in lymphomas and other cancers [41]. For example, it is well established that *miR-155* expression is induced upon Epstein-Barr virus (EBV) infection of B-cells [42]. This upregulation of *miR-155* is likely associated with EBV-induced transformation [43]. Recently, *miR-155* has been linked to EBV latency regulation, as treatment of latent EBV infected cells with a *miR-155* inhibitor reduces viral EBNA1 expression, which, in turn, reduces the EBV copy number [41]. In KSHV-infected lymphocytes, sustained expression of the KSHV *miR-155* ortholog, *miR-k12-11*, likely contributes to the increased proliferation seen in these cells [44,45]. Typically, *miR-155* is low in KSHV-infected B-cells, while *miR-k12-11* is highly expressed [44,45]. It has been demonstrated that *miR-k12-11* functionally overlaps with *miR-155*, as it is able to compensate for a lack of *miR-155* in knockout mice [46]. A miRNA located in the *MEQ* cluster of MDV-1, *mdv1-miR-M4*, is a *miR-155* ortholog [36]. Deletion or seed region mutagenesis of *miR-M4* prevents lymphoma induction in infected birds [47], suggesting a role for *miR-M4* in MDV oncogenesis. However, insertion of *miR-M4* into a related, but non-oncogenic, virus, herpesvirus of turkeys (HVT), does not result in tumor formation in infected birds [48], suggesting that other factors are needed for viral transformation. In addition to the *miR-155* ortholog, EBV encodes miRNAs sharing seed sequences with host *miR-29a/b/c*, *miR-18a/b*, *miR-520d-5p* and *miR-524-5p* [49]. Target gene analysis using an AGO pull-down assay revealed that these homologous viral/host miRNAs share many of the same targets. These conserved targets are involved in regulating apoptosis, the cell cycle and Wnt signaling. The retrovirus bovine leukemia virus (BLV) also encodes a *miR-29* ortholog, termed *blv-miR-B4* [37]. It was postulated that due to the shared targets of *miR-29*, *blv-miR-B4* may contribute to BLV-induced B-cell lymphomas. Though herpesvirus-encoded miRNAs share little sequence similarity, it has been demonstrated that several human viruses have a conserved miRNA target gene. KSHV, EBV and HCMV all encode miRNAs, which target the host gene MHC class I polypeptide-related sequence B (MICB), a stress-induced immune ligand [38]. Virally encoded miRNAs were found to downregulate MICB expression during infection. It was suggested that this miRNA-mediated downregulation of MICB is part of the herpesvirus immune evasion strategy. 

Several host miRNAs have also been linked to herpesvirus infections. The EBV encoded protein, LMP1 (latent membrane protein 1), induces the expression of host *miR-34a* upon EBV infection of human B-cells [39]. *MiR-34a* has also been found to be highly expressed in several EBV-transformed cell lines. Interestingly, *miR-34a* is considered to be a tumor suppressor miRNA, whose expression is directly regulated by p53 [50]; however, increased *miR-34a* levels are associated with enhanced growth in EBV-infected cells [39]. Two additional host miRNAs have been linked to EBV infections. The miRNAs, *miR-200b* and *miR-429*, were recently shown to be involved in the EBV lytic/latent switch [51]. Exogenous expression of *miR-200b* and *miR-429* in EBV-positive cells results in increased expression of EBV lytic genes. *MiR-200b* and *miR-429*, both members of the *miR-200* miRNA family, were shown to regulate the expression of *ZEB1* and *ZEB2*, whose gene products are transcriptional repressors involved in IL2 regulation and previously shown to regulate EBV latency [52]. In addition, EBV infection of blood-derived human B-cells results in the decreased expression of *miR-200b* [51]. In human CD34^+^ hematopoietic progenitor cells latently infected with HCMV, the host miRNA, *miR-92a*, is downregulated [53]. Decreased *miR-92a* expression results in an increase in its target gene expression, GATA-2. This increase in GATA-2 results in increased IL-10 expression, suggesting that IL-10 may be involved in HCMV latency. Knockdown of *miR-101* in HeLa cells increases HSV-1 production in infected cells [54]. *MiR-101* was shown to target the host gene, *ATP5B*, an ATP synthase, and siRNA knockdown of ATP5B expression in HeLa cells greatly reduces HSV-1 production. Analysis of host miRNA expression in porcine dendritic cells upon infection with pseudorabies virus, an alpha herpesvirus, revealed multiple differentially-expressed host miRNAs [55]. These miRNAs regulate genes and pathways with shared functions. In an MDV-transformed T-cell line, MSB1, two related host miRNAs, *miR-221* and *miR-222*, are significantly upregulated relative to splenocytes or CD4^+^ T-cells [56]. Additionally, conserved binding sites for these miRNAs are identified in the 3' UTR of *p27^kip1^*, a known *miR-221/222* target in mammals. However, the overexpression of *miR-221/222* has not been found in other MDV-transformed cell lines, suggesting these miRNAs may not play a general role in MDV transformation. Overall, both viral and cellular miRNAs are important regulators during herpesvirus infections, particularly during the lytic/latent switch and transformation. 

Polyomaviruses have also been found to encode miRNAs, though much fewer in number and variation than with herpesvirus miRNAs. Currently, all examined polyomaviruses encode a single miRNA precursor, which produces one or two mature miRNAs [28]. The majority of polyomavirus-encoded miRNAs are located antisense to the large T antigen (Tag) and function in a siRNA-like manner in the regulation of Tag expression [57,58,59,60]. Unlike herpesviruses, many polyomavirus miRNAs share sequence homology. The miRNA precursors encoded by the related polyomaviruses, bandicoot papillomatosis carcinomatosis virus type 1 and 2, are located outside of the Tag gene, but were shown to still regulate Tag expression by binding to a complementary site in its 3' UTR [60]. SV40 (simian virus 40) miRNA Tag targeting reduced the cytotoxic T-lymphocyte (CTL) response [58]. Little is known about potential host gene targets of polyomavirus miRNAs. However, recently, the host gene, *ULBP3*, a stress-induced ligand, was identified as a target of JC virus and BK virus miRNAs [58]. This targeting has been suggested to be involved in immune evasion, as it is associated with reduced natural killer cell killing of virally infected cells. No noticeable differences between a mutant murine polyomavirus lacking the miRNA precursor and wild-type virus have been observed *in vivo* [59].

Though only a few viruses, including herpesviruses, polyomaviruses, retroviruses and several invertebrate viruses, have been found to encode miRNAs, most viruses have been found to manipulate host miRNA expression (examples are given in Table 2). Several host miRNAs, *miR-28*, *miR-125b*, *miR-150*, *miR-223* and *miR-382*, are found at much higher levels in resting CD4^+^ T-cells compared to activated cells [61]. The 3' UTRs of several HIV mRNAs contain multiple binding sites for these particular miRNAs, suggesting that host miRNAs may be involved in the maintenance of HIV latency in resting CD4^+^ T-cells (Table 2, [61]). Furthermore, these miRNA binding sites are conserved between HIV strains. One symptom of hepatitis B virus (HBV)-induced hepatic cirrhosis is splenomegaly. Expression analysis revealed that 99 miRNAs are differentially expressed between normal spleens and spleens exhibiting HBV-induced hypersplenism [62], suggesting host miRNAs are associated with HBV pathogenesis. A comparison of host miRNA expression differences during infection with any of the three influenza strains, H5N1 (also known as bird flu), a reconstructed 1918 H1N1 strain (r1918) and a seasonal H1N1 virus, suggests that miRNAs are involved in influenza pathogenesis [26]. The two highly pathogenetic strains, H5N1 and r1918 altered the expression of 23 host miRNAs that are not altered by the milder seasonal H1N1 [26]. A similar study comparing cellular miRNA expression differences between r1918 and a seasonal strain (A/Texas/36/91) in mice found that over a hundred miRNAs are affected differently between the two strains [63]. Among these miRNAs are *miR-200a* and *miR-223*, whose target mRNAs are involved in the immune response and cell death pathways associated with the severe immune response found in r1918-infected lungs [63]. Microarray analysis of miRNA expression of cells infected with any one of three influenza strains of varying pathogenicity found that during early infection, *in vitro*, relatively few host miRNAs are upregulated, with only 9% of differentially-expressed miRNAs being upregulated at 24 hours post-infection (hpi) [64]. However, at 48 hpi and 72 hpi, the number of upregulated miRNAs is greatly increased, with over 90% of the differentially miRNAs being upregulated. Vaccinia virus (VACV), historically used in vaccinating against smallpox, was recently shown to induce a general downregulation of host miRNA expression in infected cells [65]. This downregulation of miRNA expression occurs within 24 hours of infection and is linked to a decrease in Dicer, a miRNA-processing enzyme, expressed in VACV-infected cells. The flavivirus, Dengue virus, also downregulates the expression of Dicer, along with other RNAi enzymes, including Drosha, AGO1 and AGO2 [66]. This reduction in RNAi proteins is linked to the viral protein, NS4B. Reduction of RNAi activity increases viral replication, suggesting that NS4B downregulation of RNAi proteins is part of the virus host defense against Dengue virus [66]. 

One of the most well-characterized examples of pathogen manipulation of host miRNAs is that of hepatitis C virus (HCV) and its use of the host liver-specific miRNA, *miR-122* [67]. Numerous profiling studies have shown that *miR-122* is highly expressed in the liver and functions in the regulation of lipid and cholesterol metabolism [68]. HCV has limited tropism *in vitro*, with very few permissive cell lines available. It was shown that a cell line permissive to HCV infection expresses high levels of *miR-122*, while non-permissive cell lines do not express *miR-122* [67]. When *miR-122* function is inhibited in the permissive cell line, HCV replication is greatly reduced. Ectopic expression of *miR-122* in non-permissive cell lines does support viral replication; however, no HCV infectious particles are produced [69]. *In silico* analysis predicted a *miR-122* binding site within the 5' UTR of the HCV genome, and mutational analysis confirmed that the binding of *miR-122* to this site enhances HCV replication [67]. It was recently shown that this enhanced viral replication is not due to a direct effect of *miR-122* on HCV RNA synthesis, as cells transfected with a *miR-122* inhibitor have lower levels of HCV RNA, but do not have reduced HCV RNA synthesis [70]. Instead, the interaction between *miR-122* and the HCV genome is likely important in stabilizing viral RNA [71].

**Table 2:** Examples of the involvement of cellular small RNAs in viral infections. HCV, hepatitis C virus; RGDV, rice gall dwarf virus; PSTVd, potato spindle tuber viroid; TSWV, tomato spotted wilt virus; CMV, cucumber mosaic virus; TEV, tobacco etch virus; TBSV: tomato bushy stunt virus.

Virus	Effector Molecule	Targets of interest	Effect on virus	Reference
EBV	*miR-155* (induced)		Associated with viral transformation	[41,42,43,44]
EBV	*miR-34a* (induced by EBV LMP1)		Enhanced viral growth	[50,51]
EBV	*miR-200b, miR-429*	*ZEB1, ZEB2*	Regulation of viral latency	[52,53]
HCMV	*miR-92a* (downregulated)	GATA-2	Increased IL-10, involved in viral latency	[54]
HSV1	*miR-101* (downregulated)	ATP5B	Enhances virus replication	[55]
MDV (MSB1 cell line)	*miR-221, miR-222*	p27^Kip1^	Associated with viral transformation	[57]
HIV	*miR-28, miR-125b, miR-150, miR-223, miR-382*	multiple HIV genes	Maintenance of latency in resting T-cells	[62]
HCV	*miR-122*	Interacts with the HCV genome	Enhances viral replication	[68,69,70]
Dengue	NS4B	Downregulates Dicer, Drosha, AGO1 and AGO2	Enhances viral replication	[67]
TSWV	Viral NS proteins	Binds to dsRNA and blocks Dicer-mediated cleavage	Disrupts antiviral response	[72]
CMV	Viral protein 2b	Binds to and inhibits the function of AGO1; also directly binds to small RNAs to block RISC function	Disrupts antiviral response	[73]
TEV	Viral helper component protease	Interacts with doubled-stranded-siRNA and prevents strand separation	Disrupts antiviral response	[74]
TBSV	Viral protein p19	Blocks RISC loading by binding to small dsRNA duplexes	Disrupts antiviral response	[75]
RGDV	pns11	Upregulation of miR-160, miR-162, miR-167, miR-168	Increased viral pathogenesis	[76]
PSTVd	*miR-396, miR-319, miR-159, miR403* (downregulated)	Transcription factors associated with plant morphology and development	Increased viroid pathogenesis	[77]

Though not as well studied as viral infections, bacterial infections have also been found to induce changes in host miRNA expression. Virulent, but not avirulent, mycobacterium inhibits TNF production by inducing the expression of host *miR-125b*, which subsequently reduces the expression of *TNF*, one of its target genes [78]. *In vitro* infection of epithelial cells with listeria alters the expression of five host miRNAs, *miR-146b*, *miR-16*, l*et-7a*, *miR-145* and *miR-155* [79]. Several of these miRNAs are known to regulate immune genes. Furthermore, the expression changes of these miRNAs vary between wild-type bacteria and less pathogenic mutant strains [79]. Taken together, these studies suggest that host miRNAs are also involved in bacterial pathogenesis. 

## 7. Counter Mechanisms to the Small RNA-Mediated Antiviral Response

In response to the siRNA-mediated antiviral response in plants, viruses have developed counter mechanisms. Examples of these mechanisms are given in Table 1. Many plant viruses encode proteins, termed viral suppressors of RNA silencing, or VSRs [80]. Recently, the non-structural proteins (NSs) of tospoviruses were shown to bind to dsRNAs, including both siRNA duplexes and miRNA/miRNA* molecules [81]. The interaction between tomato spotted wilt virus (TSWV) NSs and dsRNA was shown to block Dicer-mediated cleavage. In addition, the binding of NSs to mature siRNA, after Dicer processing, also blocks this form of antiviral response. The 2b protein of cucumber mosaic virus (CMV), a cucumovirus, possesses the ability to bind to and inhibit AGO1, thus blocking RISC function [72]. Furthermore, similar to tospovirus NSs, 2b can also directly interact with small RNAs, suggesting that 2b employs multiple mechanisms to block antiviral RNA silencing. The helper component protease of tobacco etch virus (TEV) is able to interact with ds-siRNA and prevent strand separation [73]. The p19 protein of tomato bushy stunt virus (TBSV) blocks RISC loading by binding small dsRNA duplexes [74]. It was recently been shown, however, that some Nicotiana plants have developed counter mechanisms to p19 RISC interference [75]. In addition to altering siRNA generation, plant VSRs can also alter host miRNA expression. Rice gall dwarf virus (RGDV), a phytoreovirus, encodes a VSR, termed *Pns11* [82]. When Pns11 is individually expressed in rice, the plants display phenotypic characteristics reminiscent of RGDV-infected plants. Transgenic Pns11-expressing plants have altered levels of four host miRNAs, *miR-160*, *miR-162*, *miR-167* and *miR-168*. The altered expression of these miRNAs, in particular, the increased expression of *miR-167,* correlates to the disease phenotype. It has been suggested that alterations in the host gene targets of these differentially-expressed miRNAs contributes to RGDV pathogenesis. For example, the expression of a *miR-167* target gene, *ARF8*, is altered in Pns11 transgenic plants [73]. Recently, it has been demonstrated that co-infections of plant viruses can result in more severe alterations in miRNA expression [76]. Tobacco plants co-infected with potato virus X (PVX) and either potato virus Y (PVY) or plum pox virus (PPV) exhibit up to a 14-fold difference in host miRNA expression compared to mock or singularly-infected plants. These differentially-expressed miRNAs target genes are involved in stress responses, among others, suggesting that the more dramatic alterations in host miRNA expression may be linked to the increased symptom severity of co-infected plants. 

Viroids are a class of short non-coding circular single-stranded RNA (ssRNA), which infect plants and can cause disease symptoms, including growth retardation and necrosis [83]. Viroids rely entirely on host machinery for replication. As with viral infections, plants often employ siRNA-mediated immunity in response to viroid infection [84,85]. In viroid-infected grapevine tissues, multiple viroid-derived sRNAs of 21 nt, 22 nt and 24 nt have been found [84]. The fact, that sRNAs of varying lengths have been identified suggests that multiple DCLs, and, therefore, RNA silencing mechanisms, are involved in the host defense against viroids. For example, 60 distinct siRNA species generated from the peach latent mosaic viroid (PLMVd) ranging in size from 20 nt to 26 nt have been identified and are equally distributed between (+) and (−) polarities [85]. Rutgers tomato plants infected with the potato spindle tuber viroid (PSTVd) strain AS1 show disease symptoms, including necrosis and dwarfism [86]. Analysis of host small RNA expression patterns revealed that several host miRNAs, *miR-159*, *miR-396*, *miR-319* and *miR-403*, are downregulated in PSTVd infected plants [86]. These miRNAs are known to regulate transcription factors involved in plant morphology and development, suggesting that changes in host miRNA expression and function are associated with viroid pathogenesis. 

## 8. Small RNAs in Prokaryotic Immunity

Recently, an RNAi-like defense system has been identified in prokaryotes, both bacteria and Archaea, which consists of clustered regularly-interspaced short palindromic repeats (CRISPR) and their associated (Cas) proteins [77]. The CRISPR-Cas system is considered analogous to the vertebrate adaptive immune response, as it functions in memory-based defense [87]. In CRISPR-based immunity, regions of an invading phage or plasmid, termed protospacers, are acquired and incorporated into the bacterial genome at CRISPR loci and are now called spacers [88]. It has been shown that protospacers are not randomly selected [89], but rather, there is a propensity for specific motifs in the invading DNA, such as an AAG protospacer-adjacent motif (PAM) in protospacer regions [90]. A CRISPR locus consists of a 5' leader, which functions as a recognition site for spacer incorporation [91]. The leader sequence is followed by an array spacer and repeat sequences [91]. After assembly, a precursor CRISPR RNA consisting of the leader sequence is followed by the spacer/repeat regions [92]. This precursor RNA is then processed into mature crRNA [90]. CrRNA recognize the targeted protospacer regions in foreign nucleic acid molecules, either DNA or RNA, which then leads to their degradation by specific Cas proteins [92]. CrRNA expression can be both continual and induced by the detection of foreign DNA [93]. It has been suggested that, at least in *E. coli*, interaction between crRNA and protospacer regions in foreign DNA can lead to a priming interaction, resulting in the attainment of new spacers [90]. 

Currently, three major CRISPR-Cas types, I, II and III, have been identified based on the specific Cas proteins involved in the targeted degradation of foreign nucleic acid [94]. Each CRISPR-Cas type is further divided into various subtypes. It appears that there is a plasmid/phage-specific preference in which the CRISPR-Cas subtype is activated. For example, it was recently demonstrated that *sulfolobus islandicus* rod-shaped virus 2 (SIRV2) infection of *Sulfolobus islandicus* preferentially induces CRISPR-Cas subtype III-B over other subtypes, such as I-A and I-D, present in the *S. islandicus* genome [95]. This suggests that the CRISPR-Cas system is a large and diverse acquired defense mechanism in prokaryotes. Just as eukaryotic viruses have developed several anti-RNAi defense mechanisms, it was recently shown that bacteriophages have developed anti-CRISPR strategies [96]. Genomic analysis of *Pseudomonas aeruginosa*-infecting phages identified five genes associated with CRISPR-Cas resistance [96]. Mutation of these genes leads to increased susceptibility of the phages to crRNA degradation. It is likely that similar anti-CRIPSR mechanisms in other bacteriophages also exist.

## 9. Conclusions

Though often through different mechanisms, small RNAs are major players in host defense and pathogen manipulation in both plants and animals (Table 1). The fact that small RNAs can alter the expression of their target genes to varying degrees makes them ideal modulators of immunity. If an immune response is too robust, it can be detrimental for the host, on the other hand, if the response is not potent enough, then the pathogen cannot be properly suppressed. Small RNAs, therefore, provide eukaryotes with the ability to fine-tune the immune system to obtain the optimal response. Pathogens, in turn, have evolved numerous ways in which to commandeer the small RNA-mediated regulatory system. Small RNAs provide pathogens with an ideal method in which to manipulate host gene expression, without triggering an anti-pathogenic response. 
